# Zoonotic necrotizing myositis caused by *Streptococcus equi* subsp. *zooepidemicus* in a farmer

**DOI:** 10.1186/s12879-017-2262-7

**Published:** 2017-02-15

**Authors:** Bård Reiakvam Kittang, Veronika Kuchařová Pettersen, Oddvar Oppegaard, Dag Harald Skutlaberg, Håvard Dale, Harald G. Wiker, Steinar Skrede

**Affiliations:** 1 Department of Medicine, Haraldsplass Deaconess Hospital, Bergen, Norway; 2 The Gade Research Group for Infection and Immunity, Department of Clinical Science, University of Bergen, Bergen, Norway; 3 Department of Medicine, Haukeland University Hospital, Bergen, Norway; 4 Department of Microbiology, Haukeland University Hospital, Bergen, Norway; 5 Department of Orthopaedic Surgery, Haukeland University Hospital, Bergen, Norway

**Keywords:** Case report, Zoonosis, *Streptococcus equi* subsp. *zooepidemicus*, Necrotizing myositis

## Abstract

**Background:**

*Streptococcus equi* subsp. *zooepidemicus* is a beta-hemolytic group C streptococcus mainly causing infections in domesticated animals. Here we describe the first case of zoonotic necrotizing myositis caused by this bacterium.

**Case presentation:**

The patient was a 73-year-old, previously healthy farmer with two asymptomatic Shetland ponies in his stable. After close contact with the ponies while feeding them, he rapidly developed erythema of his left thigh and sepsis with multiple organ failure. The clinical course was severe and complicated, requiring repetitive surgical excision of necrotic muscle, treatment with vasopressors, mechanical ventilation and continuous venovenous hemofiltration, along with adjunctive hyperbaric oxygen therapy. The patient was discharged from hospital at day 30, without obvious sequelae.

The streptococcal isolate was identified as *Streptococcus equi* by MALDI-ToF MS, and was later assigned subspecies identification as *S. equi* subsp. *zooepidemicus*. Multilocus sequence typing identified the strain as a novel sequence type (ST 364), closely related to types previously identified in horses and cattle. A focused proteomic analysis revealed that the ST 364 expressed putative virulence factors similar to that of *Streptococcus pyogenes*, including homologues of the M protein, streptodornases, interleukin 8-protease and proteins involved in the biosynthesis of streptolysin S.

**Conclusion:**

This case illustrates the zoonotic potential of *S. equi* subsp. *zooepidemicus* and the importance of early clinical recognition, rapid and radical surgical therapy, appropriate antibiotics and adequate supportive measures when necrotizing soft tissue infection is suspected. The expression of *Streptococcus pyogenes*-like putative virulence determinants in ST 364 might partially explain the fulminant clinical picture.

**Electronic supplementary material:**

The online version of this article (doi:10.1186/s12879-017-2262-7) contains supplementary material, which is available to authorized users.

## Background

*Streptococcus equi* subsp. *zooepidemicus* (*S. zooepidemicus*) is a beta-haemolytic group C streptococcus able to colonize the upper airways of horses and produce diverse clinical manifestations in domesticated animals, including respiratory tract infections, mastitis and meningitis [1–4]. *S. zooepidemicus* rarely causes human infection, and the mechanism is supposed to be zoonotic transmission by direct contact with infected or colonized animals or the consumption of unpasteurized milk products [5–8]. This streptococcus has been associated with a wide range of severe human infections, including cellulitis, pericarditis, toxic shock syndrome, endovascular infections, pneumonia, septicaemia, meningitis, arthritis and spondylodiscitis [5, 9–13]. It has also caused a large outbreak of post-streptococcal glomerulonephritis in Brazil [14].

Necrotizing myositis is a very rare and potentially lethal infection, constituting the most severe form of necrotizing soft tissue infections (NSTI). Monomicrobial NSTI is most often caused by *Streptococcus pyogenes* (*S. pyogenes*), and frequently associated with septic shock and high mortality rates [15–17]. NSTI caused by *S. zooepidemicus* was recently documented in a dog shortly after subcutaneous vaccination [18], but to our knowledge, human NSTI caused by *S. zooepidemicus* has not been reported previously.

Here we present a case of necrotizing myositis caused by *S. zooepidemicus* in a farmer who was in close contact with his two Shetland ponies prior to the infection.

## Case presentation

A 73 –year-old male patient was transmitted with air ambulance from a local hospital to Haukeland University Hospital (HUH) in western Norway with septic shock and clinical suspicion of NSTI in his left thigh.

His previous medical history included paroxysmal atrial fibrillation, treated with flecainide, and psoriatic arthritis. He was a farmer, with two Shetland ponies in his stable. A few days prior to hospitalization, he had acquired minor abrasions and blisters on his fingers and subsequently been in direct contact with the ponies upon feeding them.

In the afternoon on day 1, he was admitted to the local hospital with acute pain in his left groin. He rapidly developed symptoms and signs of septic shock. Blood cultures were drawn, and empiric antibiotic therapy with penicillin G, clindamycin and gentamicin was initiated. Computed tomography imaging of the pelvis and left thigh showed possible necrotizing myositis or pyomyositis and he was thereupon rapidly transferred to a tertiary care facility.

Upon admission at HUH day 2 the patient was intubated and maintained an adequate blood pressure of 139/61 mmHg on a low-dose noradrenaline-infusion (0.03 μg/kg/min). His temperature was 38.7 ° C and the pulse rate was 108 per minute. A relatively sharply demarcated erythema in the left thigh was observed, but neither bulla, ecchymosis or skin necrosis was present. Blood cultures and initial blood chemistry analyses were obtained. Further diagnostic and therapeutic strategies were discussed in a multidisciplinary team consisting of an orthopaedic and plastic surgeon, infectious disease consultant and anaesthesiologist. A preoperative magnetic resonance tomography was rapidly performed, revealing probable necrotizing fasciitis and myositis primarily involving the adductor muscles and *musculus pectineus* in the left thigh. The patient was then transmitted to the operation theatre with a rapidly spreading erythema and a fulminant septic shock, now requiring high-dose noradrenaline infusion (0.3 μg/kg/min). Initial blood chemistry results were as follows, with normal range values in parentheses:

C-reactive protein 129 mg/l (<5 mg/l); leucocytes 1.5 × 10^9^/l (3.5 × 10^9^/l to 11.0 × 10^9^/l); neutrophils 1.2 × 10^9^/l (1.7 × 10^9^/l to 8.2 × 10^9^/l); haemoglobin 12.4 g/dl (13.4 to 17.0 g/dl); thrombocytes 85 × 10^9^/l (145 × 10^9^/l to 348 × 10^9^/l); creatinine 63 μmol/l (60 μmol/l to 105 μmol/l); myoglobin 10,323 μmol/l (<70 μmol/l), creatine kinase 9550 U/l (40 U/l to 80 U/l); bilirubin 20 μmol/l (<19 μmol/l); activated partial thromboplastin time 47 s (30 to 44 s); International Normalized Ratio 1.3 (<1.1), lactate 4.8 mmol/l (0.9 mmol/l to 1.7 mmol/l), procalcitonin 23.2 μg/l (<0.1 μg/l).

At the first surgical exploration extensive muscle necrosis was found, requiring excision of *m. adductor longus*, along with the anterior part of *m. adductor magnus*. Profound subcutaneous exudation (“dishwater fluid”), but not frank pus, was also observed, compatible with the diagnosis necrotizing myositis. Perioperative tissue and fluid samples (*n* = 8) were obtained for microbiological and histopathological analyses, and a rapid microscopic evaluation of a Gram stained smear from the site of infection revealed Gram positive diplo- and streptococci. The patient was transmitted to the intensive care unit, with a tentative diagnosis of streptococcal necrotizing myositis.

On day 3 cultures from blood, tissue and fluid grew beta-haemolytic colonies on blood agar. Species identification of the bacterial isolate as *Streptococcus equi* was performed using matrix-assisted laser desorption ionization-time of flight mass spectrometry (MALDI-ToF MS), using Microflex™ with the MALDI Biotyper database (Bruker Daltonik, Bremen, Germany) and subsequently group C carbohydrate specificity was determined using a slide agglutination test (Oxoid, Cambridge, United Kingdom). The group C streptococcus was fully susceptible to all tested antibiotics, with the following MIC-values: penicillin G 0.016 mg/l, ceftriaxone 0.064 mg/l and clindamycin 0.25 mg/l. In order to obtain a correct subspecies identification, the streptococcal isolate was sent to the national reference laboratory at the Norwegian Veterinary Institute in Oslo for further analyses.

The patient still required treatment with vasopressors and mechanical ventilation, and continuous venovenous hemofiltration was started due to an elevated level of creatinine, oliguria and hyperkalaemia. Gentamicin was discontinued, and therapy with penicillin G and clindamycin was sustained. A second surgical revision was performed, revealing progressive necrosis of the subcutaneous tissue and muscle of the left thigh that was treated with a resection of almost the entire *m. adductor magnus* and *brevis*, *m. pectineus* and *m. gracilis.*

The further clinical course was severe and complicated, characterized by a need for repeated surgical excision of necrotic tissue, hyperbaric oxygen (HBO) therapy and sustained intensive care treatment of gradually resolving organ dysfunctions and a nosocomial soft tissue superinfection probably caused by *Pseudomonas aeruginosa*, which grew from a wound specimen upon clinical deterioration on treatment with penicillin G and clindamycin.

**Fig. 1:**
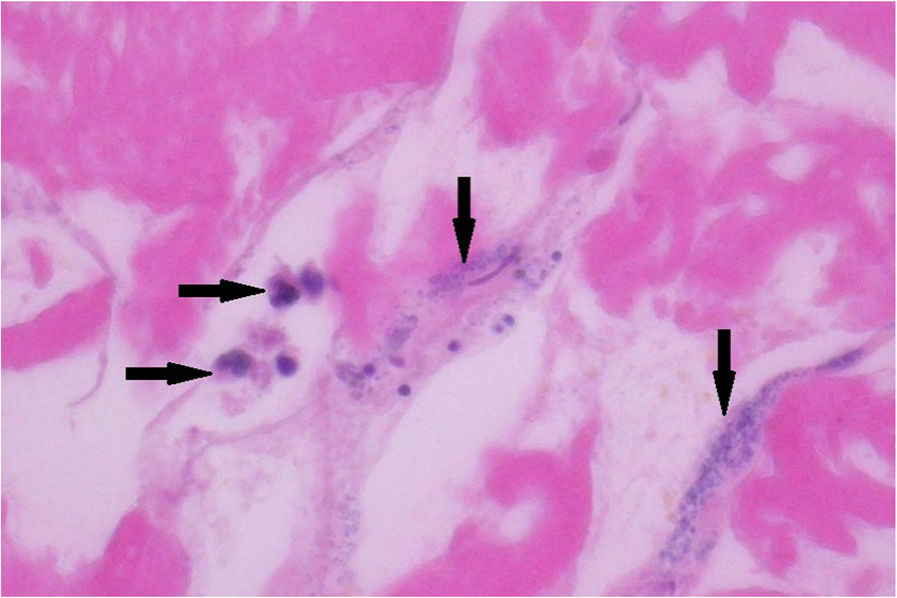
Histological preparation of muscle biopsy (Hematoxylin-eosin staining, × 40), showing infiltration of neutrophil granulocytes (*rightward arrows*) and aggregates of streptococcal bacteria (*downward arrows*), surrounded by necrotic muscular tissue

Table 1 highlights important aspects of the clinical course and summarizes the major microbiological findings, and Fig. 1 shows the muscular necrosis, infiltration of granulocytes and streptococci found upon histopathological analyses.

**Table 1:** Summary of the clinical, microbiological and histopathological findings

	Day 2	Day 3	Day 4	Day 5	Day 6	Day 7	Day 8	Day 9	Day 11	Day 14
Surgery	Excision of necrotic tissue	Excision of necrotic tissue	Excision of necrotic tissue	Excision of necrotic tissue	Inspection and wound debridement		Inspection and wound debridement		Surgical closure	
Antibiotics	Pen G + Clinda + Genta	Pen G + Clinda + Gentamicin	Pen G+ Clinda	Pen G + Clinda	Pen G + Clinda	Pen G + Clinda	Pen G + Clinda	Clinda + Cefta+ Cipro	Pen G + Clinda+ Cipro	Pen G + Cipro
Microbiology and pathology		Streptococci isolated from blood and tissue. Histo-pathological findings compatible with necrotizing myositis	Confirmed species-identifi-cation of *S. equi.*					*P. aeruginosa*, *Bacteroides* spp. and *S. equi* from infected soft tissue		Confirmed subspecies-identification of *S. equi.* subsp. *zooepidemicus*
Organ dysfunction^a^	Circulatory Respiratory Coagulation	Circulatory Respiratory Renal Coagulation	Circulatory Respiratory Renal Coagulation	Circulatory Respiratory Renal Coagulation	Respiratory Renal Coagulation	Respiratory Renal Coagulation	Respiratory Renal Coagulation	Respiratory Renal Coagulation	Respiratory Coagulation	
SOFA-score^b^		11	13	14	13	7	9	5	3	
Vasopressors	NA	NA + Vasopressin	NA + Vasopressin	NA + Vasopressin						
HBO-therapy			Yes	Yes						
CVVHF			Yes	Yes						
Respirator	Yes	Yes	Yes	Yes	Yes	Yes	Yes	Yes	Yes	

*Pen G* penicillin G, *Clinda* clindamycin, *Genta* gentamicin, *Cefta* ceftazidim, *Cipro.* ciprofloxacin*, NA* noradrenaline, *HBO* hyperbaric oxygen, *CVVHF* continuous venovenous hemofiltration, + plus

^a^ Organ dysfunction assessment according to Sequential Organ Failure (SOFA)-score

^b^ SOFA-score was not performed on day 2 and day 14

On day 11, the tracheostomy was removed. The patient was then transferred from the ICU to the infectious disease ward at HUH and further on to the local hospital at day 20. He was treated with penicillin and ciprofloxacin until day 30, and was discharged without any signs of systemic organ dysfunction.

Thereafter, he received physiotherapy on an outpatient basis for a short period and quickly regained adequate muscular function. Twelve months after discharge from hospital he was feeling well, worked full-time and went hiking in the local mountains on a regular basis.

## Molecular analyses

The strain was identified as *S. zooepidemicus* according to standard microbiological procedures at the Norwegian Veterinary Institute [19]. In order to further identify the strain on a molecular level we performed multilocus sequence typing (MLST), as previously described, with primer pairs targeting seven housekeeping genes [20]. The sequence analyses showed that this particular strain belonged to a new sequence type of *S. zooepidemicus*, ST 364, closely related to sequence types previously recovered from horses and cattle (http://pubmlst.org/szooepidemicus).

In order to explore potential virulence determinants of ST 364, a proteomic analysis based on liquid chromatography-tandem mass spectrometry (LC-MS/MS) was performed (Additional files 1 and 2). The analysis was primarily based on a selection of putative virulence factors identified in the only published *S. zooepidemicus* genome associated with human infection [21], with a particular focus on a search for homologues of selected well-known virulence factors in *S. pyogenes.*

The proteomic analysis identified altogether 18 proteins linked to *S. zooepidemicus* virulence (Table 2). For all of these proteins we had derived their relative amounts by employing the proteomic software MaxQuant, which allows for proteins label-free quantification (LFQ) [22]. As a reference protein served elongation factor TU, which is one of the most abundant proteins in most bacterial cells [23]. A number of virulence factors were detected at relatively high levels in ST 364 under *in vitro* culturing condition. These were cell-surface M-like protein SzM, streptodornase SdzB, serine protease ScpC/CepA, and several proteins involved in streptolysin S biosynthesis (SagCDG). In addition, the analysis identified three other proteins engaged in streptolysin S biosynthesis (SagBHI), a putative peptidoglycan hydrolase (GbpB/SagA/PcsB), several surface-anchored proteins (MlpZ, Fbp, SpaZ and Szp) and enzymes that assist in breaking down the host connective tissue (HylZ) and activate plasminogen (Skc_1, Skc_2).

**Table 2:** Identification of *Streptococcus equi subsp. zooepidemicus* ST 364 putative virulence factors by proteomic analysis

Protein name	Gene name(s)	Relative cellular abundance^a^	Relative quantification^b^ (log_2_ LFQ intensity)	
			Median	Standard deviation
Fibronectin/fibrinogen-binding protein	*fbp*	+	21.7	0.1
Beta-N-acetylglucosaminidase/ Hyaluronidase	*hylZ*	+	20.7	0.4
M-like protein	*mlpZ*	++	24.9	0.2
Secreted antigen GbpB/SagA/PcsB, putative peptidoglycan hydrolase	Sez_0018	+	21.0	0.1
Streptolysin S biosynthesis protein SagB	*sagB*	+	20.7	0.1
Streptolysin S biosynthesis protein SagC	*sagC*	++	26.1	0.1
Streptolysin S biosynthesis protein SagD	*sagD*	++	25.4	0.1
Streptolysin S export protein SagG	*sagG*	++	26.8	0.1
Streptolysin S export transmembrane permease SagH	*sagH*	+	23.1	0.1
Streptolysin S export transmembrane permease SagI	*sagI*	+	22.3	0.1
Serine endopeptidase, lactocepin, interleukin-8 protease-like protein	*scpC/cepA*	++	25.1	0.1
Streptodornase type B	*sdzB*	++	26.6	0.2
Streptodornase type D	*sdzD*	++	24.1	0.1
Streptokinase; Skc_1 protein	*skc_1*	++	24.3	0.1
Uncharacterized streptokinase-like protein; Skc_2 protein	*skc_2*	+	23.7	0.1
Protective antigen-like protein, fibrinogen-and Ig-binding protein	*spaZ*	+	23.7	0.1
Antiphagocytic cell surface-anchored fibrinogen-and IgG Fc-binding protein SzM	*szm*	+++	31.4	0.1
Fibrinogen-binding cell surface-anchored protein SzP	*szp*	+	22.0	0.3
Elongation factor Tu^c^	*tuf*	+++	31.9	0.1

^a^ An arbitrary scale of proteins cellular amounts based on the range of log_2_ LFQ intensities (between 19.6 and 34.0) within the sample: +++ highly abundant protein (log_2_ LFQ intensity: 29–34), ++ protein present in moderate amounts (24–28), + low abundant protein (19–23)

^b^ Label-free quantitative LFQ intensity of a protein is proportional to the quantity of the protein in the sample

^c^ Reference protein with high cellular abundance

## Discussion

This first report of zoonotic necrotizing myositis caused by *S. zooepidemicus* illustrates the crucial role of a multidisciplinary approach at admission, rapid clinical identification, early and repeated surgery, adequate supportive measures and appropriate antibiotics in the treatment of NSTI. The findings from a previous study on 89 cases of NSTI showed that time to surgery is an important prognostic factor [24], and it is likely that early surgery and meticulous surgical follow-up was a prerequisite for therapeutic success in our case. The empiric antibiotic treatment consisted of penicillin, clindamycin and gentamicin, in accordance with the national antibiotic guidelines in Norway (http://sites.helsedirektoratet.no/sites/antibiotikabruk-i-sykehus/Sider/default.aspx).

When the bacterial cause was identified and the antibiotic susceptibility pattern was confirmed, the patient was further treated with a combination of penicillin and clindamycin. Clindamycin has been shown to be superior to beta-lactam-antibiotics in two observational studies on streptococcal NSTI, and furthermore, to reduce mortality of severe GAS infections including toxic shock and NSTI [25–28]. Hence, although ST 364 did not belong to *S. pyogenes*, the combination of penicillin and clindamycin appeared to be the most sensible antibiotic regimen, in line with the recommendations for treatment of beta-haemolytic NSTI in the IDSA-guidelines [29].

The potential effect of HBO - therapy in NSTI has mainly been evaluated in small, retrospective studies, including patients with varying disease severity and a wide range of different bacteriological aetiologies, and the results are diverging [30–32]. The findings from a recent investigation, however, indicate that the most severely affected NSTI-patients might benefit the most from HBO-therapy [33]. Our patient had life-threatening sepsis with multiorgan-dysfunction and extensive muscle necrosis, and received HBO-therapy on two consecutive days. We believe that prompt and radical surgery along with appropriate antibiotics were the treatment cornerstones in this case, but it is conceivable that HBO-therapy might have contributed to the relatively rapid improvement of the infection.

Zoonotic transmission to man from asymptomatic horses colonized with *S. zooepidemicus* in the upper airways has previously been described [8]. Unfortunately, nasopharyngeal swabs were not obtained from the healthy ponies in the present case. The patient developed sores and abrasions on his fingers prior to the infection, was in direct contact with the ponies while feeding them, and had no direct contact with other animals before and around the time of infection. Hence, a direct transmission of ST 364 from pony to human is suspected and also supported the close genetic relationship between ST 364 and other *S. zooepidemicus* sequence types from horses, namely ST 40, ST 138 and ST 214 (http://pubmlst.org/szooepidemicus).

According to a genomic study on animal isolates of *Streptococcus equi* subspecies *equi* and *zooepidemicus*, the former probably has evolved from an ancestral *S. zooepidemicus* into a specialized pathogen primarily responsible for strangles in horses [34]. Furthermore, although *S. zooepidemicus* is able to cause significant respiratory tract infections in horse, they are occasionally associated with asymptomatic nasopharyngeal carriage [1, 35]. Both *S. equi* subsp. *equi* and *zooepidemicus* share extensive homology with *S. pyogenes*, and lateral genetic exchange between these three streptococcal species has been inferred [34]. In a genomic study on a *S. zooepidemicus* strain responsible for an outbreak of post-streptococcal glomerulonephritis, the majority of the putative virulence determinants were *S. pyogenes*-homologues [21]. Moreover, like *S. pyogenes*, *S. zooepidemicus* causing human infection tend be associated with a wide range of severe clinical manifestations [5, 9–13].

Knowledge on virulence determinants of severe *S. zooepidemicus*-infections is scarce, particularly in humans. In the aforementioned genomic study by Beres et al., approximately 100 genes homologous to putative or proven virulence factors in other bacteria, primarily *S. pyogenes*, were identified, including genes encoding factors involved in adhesion, immune response evasion, host cell cytotoxicity, bacterial dissemination and mitogenicity [21]. The highlighted proteomic data on ST 364 presented in this study, indicate a marked expression of homologues of virulent determinants (shown in parenthesis) known to play a role in the pathogenesis of beta-haemolytic streptococcal NSTI, namely the M protein (SzM), streptodornases (SdzB), interleukin 8-protease (SCpC/CepA), and proteins involved in the streptolysin S biosynthesis (sag C/D/G) [36–39]. Notably, we could not find evidence for superantigen activity in ST 364, in concordance with in a recent study on horses, where only 50% of the *S. zooepidemicus*-isolates contained superantigen-encoding genes [40].

Our molecular data does not allow for firm conclusions on the virulence properties of ST 364, and call for a more thorough genetic and proteomic dissection of this particular strain. Furthermore, although our patient did not have any obvious susceptibility for severe streptococcal disease, the clinical outcome was probably a result of a complex interplay between host factors and bacterial virulence.

Taken together, we speculate that this case story matches the available microbiological, molecular and clinical data on *S. zooepidemicus* quite well:

We suspect that a *S. zooepidemicus*-strain, perhaps not fit do produce clinically significant nasopharyngeal infection in the ponies, but potentially armed with virulence properties homologous to those of *S. pyogenes*, was transmitted to a human without known predisposition to infection, causing a severe *S. pyogenes*-like clinical picture.

## Conclusion

This first case report on necrotizing myositis caused by *S. equi* subsp*. zooepidemicus* illustrates the zoonotic potential and clinical versatility of this beta-haemolytic streptococcus. It is also a reminder of the fulminant course streptococcal NSTI can pursue, requiring prompt recognition, extensive surgery, appropriate antibiotics and supportive treatment in the intensive care unit.

The strain ST 364 belonged to a new sequence type closely related to *S. zooepidemicus*-strains previously identified in horses, and expressed *S. pyogenes*-like putative virulence determinants.

## Key points

- *S. zooepidemicus* is primarily an animal pathogen, but occasionally fit to produce severe zoonotic infections, including the exceedingly rare manifestation necrotizing myositis
- Necrotizing myositis requires prompt clinical recognition along with adequate surgical, antibiotic and supportive therapy
- The *S. pyogenes*-like, fulminant course of necrotizing myositis caused by *S. zooepidemicus* might be partially explained by homologous virulence properties

## Additional files

Additional file 1: Proteomic analysis – materials and methods. Methodology of sample preparation for LC-MS/MS analysis and the MS/MS data analysis. (DOC 40 kb)
Additional file 2: Data sheets – Proteins and Peptides. Results from the proteomic analysis; *Streptococcus* *equi *subsp. *zooepidemicus* proteins detected at 1% FDR and corresponding peptide sequences detected at 1% FDR. (XLSX 45 kb)

## Abbreviations

CVVHF: Continuous venovenous hemofiltration
HBO: Hyperbaric oxygen
HUH: Haukeland University Hospital
LC-MS/MS: Liquid chromatography-tandem mass spectrometry
LFQ: Label-free quantification
MALDI-ToF MS: Matrix-assisted laser desorption ionization-time of flight mass spectrometry
MIC: Minimal inhibitory concentration
MLST: Multilocus sequence typing
NA: Noradrenaline
NSTI: Necrotizing soft tissue infections
*S. pyogenes*: *Streptococcus pyogenes*
*S.zooepidemicus*: *Streptococcus equi subsp. zooepidemicus*
