# Supplementary material for: Zoonotic necrotizing myositis caused by Streptococcus equi subsp. zooepidemicus in a farmer
Source: BMC Infect Dis. 2017 Feb 15;17:147. doi: 10.1186/s12879-017-2262-7 (PMC5312586; doi:10.1186/s12879-017-2262-7)
Supplement: Additional file 1: — Proteomic analysis – materials and methods. Methodology of sample preparation for LC-MS/MS analysis and the MS/MS data analysis. (DOC 40 kb) [file 12879_2017_2262_MOESM1_ESM.doc]

Additional file 1

Proteomic analysis – materials and methods

Cells of the clinical *S. zooepidemicus* isolate were in 4 independent biological replicates cultured on blood agar, harvested, washed and lysed according to a protocol that was previously described for uropathogenic *E. coli* . The whole cell lysates were processed as described earlier, according to the Multiple Enzymes for sample Digestion – Filter-Aided Sample Preparation (MED-FASP) protocol . In short, the cell lysates were treated with trypsin and LysC in a two-step digestion reaction, and the resulting peptide mixtures were desalted and lyophilized. Prior to the liquid chromatography-tandem mass spectrometry (LC-MS/MS) analysis, the peptides were resuspended in 0.1% formic acid and 2% acetonitrile. The LC -MS/MS analysis was carried out on an Ultimate 3000 RSLC system (Thermo Scientific, Waltham, MA, USA) connected to a linear quadrupole ion trap (LTQ)-Orbitrap mass spectrometer (Thermo Scientific) equipped with a nanoelectrospray ion source. The settings of the LC and MS/MS were identical as previously described . All MS raw data files were processed together in MaxQuant (version 1.5.3.28) . Andromeda search engine integrated in the MaxQuant framework performed the spectra search against a selection of known and putative *Streptococcus equi* subsp*. zooepidemicus* virulence proteins downloaded from the UniProtKB database (1,660 entries, 4th July 2016). Enzyme specificity was defined in group-specific parameters as either to trypsin or LysC, allowing N-terminal cleavage to proline. The spectra of the LysC and tryptic fractions originating from the same replicate were combined in MaxQuant. Standard settings were used for MaxQuant searches, except that lysine acetylation and glutamate/ glutamine conversion to pyro-glutamate were set as variable modifications in addition to N-terminal acetylation and methionine oxidation. The “match between runs” option was enabled to match spectral identifications across different replicates. Normalized spectral proteins intensities (label-free quantification [LFQ] intensity) were derived by the MaxLFQ algorithms . The MaxQuant output data were analyzed with the Perseus module ([http://www.perseus-framework.org](http://www.perseus-framework.org/), version 1.5.1.6).

**Reference list**

1. Pettersen VK, Mosevoll KA, Lindemann PC, Wiker HG: **Coordination of Metabolism and Virulence Factors Expression of Extraintestinal Pathogenic Escherichia coli Purified from Blood Cultures of Patients with Sepsis**. *Molecular & cellular proteomics : MCP* 2016, **15**(9):2890-2907.

2. Wisniewski JR, Mann M: **Consecutive proteolytic digestion in an enzyme reactor increases depth of proteomic and phosphoproteomic analysis**. *Analytical chemistry* 2012, **84**(6):2631-2637.

3. Cox J, Mann M: **MaxQuant enables high peptide identification rates, individualized p.p.b.-range mass accuracies and proteome-wide protein quantification**. *Nature biotechnology* 2008, **26**(12):1367-1372.

4. Cox J, Hein MY, Luber CA, Paron I, Nagaraj N, Mann M: **Accurate proteome-wide label-free quantification by delayed normalization and maximal peptide ratio extraction, termed MaxLFQ**. *Molecular & cellular proteomics : MCP* 2014, **13**(9):2513-2526.
